# Supplementary material for: Locomotor recovery following contusive spinal cord injury does not require oligodendrocyte remyelination
Source: Nat Commun. 2018 Aug 3;9:3066. doi: 10.1038/s41467-018-05473-1 (PMC6076268; doi:10.1038/s41467-018-05473-1)
Supplement: Supplementary file 1 — Supplementary Information [file 41467_2018_5473_MOESM1_ESM.pdf]

# Supplementary Figures

Locomotor recovery following contusive spinal cord injury does not require oligodendrocyte remyelination

*Duncan et al.*

**Title: Locomotor recovery following contusive spinal cord injury does not require oligodendrocyte remyelination**

**Authors:** Greg J. Duncan[#,1,2], Sohrab B. Manesh[#,1,3], Brett J. Hilton[1,2,4], Peggy Assinck[1,3], Jie Liu[1], Aaron Moulson[1,2], Jason R. Plemel[5] and Wolfram Tetzlaff[1,2,6,*].

**Affiliations:** [1] International Collaboration on Repair Discoveries (ICORD), University of British Columbia (UBC). 818 West 10th Avenue, V5Z 1M9, Vancouver BC, Canada.

[2] Department of Zoology

[3] Graduate Program in Neuroscience

[4] Current address: Deutsches Zentrum für Neurodegenerative Erkrankungen (DZNE). Sigmund-Freud-Straße 27, 53127 Bonn, Germany

[5] The department of Clinical Neurosciences, Hotchkiss Brain Institute, University of Calgary. 3330 Hospital Drive NW, T2N 4N1, Calgary, Alberta, Canada.

[6] Department of Surgery

[#] These authors contributed equally to this work

*** Corresponding Author:**
Wolfram Tetzlaff, MD, PhD.
John and Penny Ryan British Columbia Leadership Chair in Spinal Cord Research
Professor, Departments of Zoology and Surgery
Director ICORD (International Collaboration on Repair Discoveries)
University of British Columbia
Blusson Spinal Cord Centre
818 West 10th Avenue
Vancouver, BC, V5Z 1M9
*Phone:* 604 675 8848
*Fax:* 604 675 8820
*Email:* tetzlaff@icord.org

**Supplementary File Includes:**

Supplementary Figures 1-3

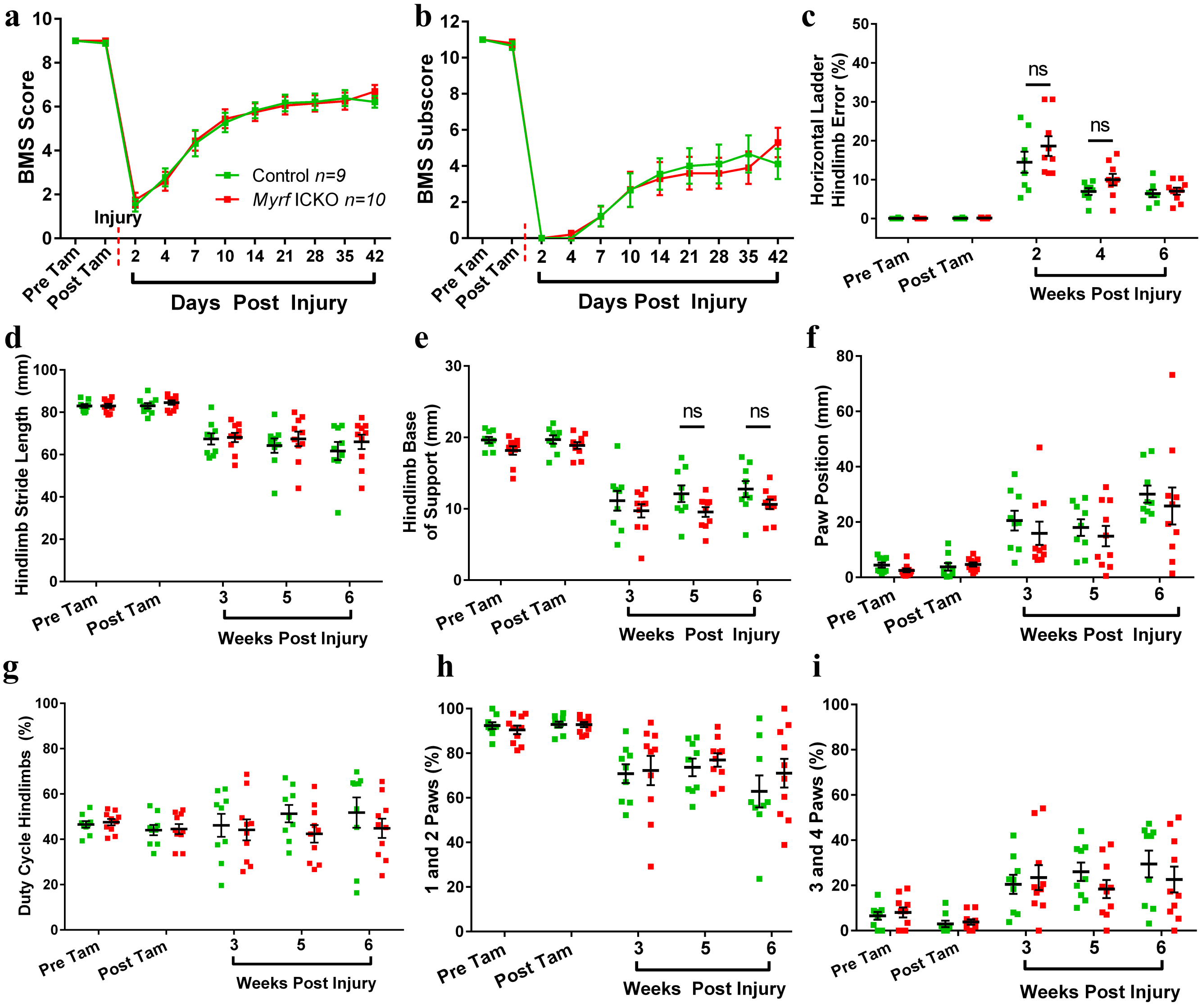


**Supplementary Fig. 1**

An additional cohort of control and *Myrf* ICKO mice demonstrating again no difference in recovery of hindlimb motor function following moderate thoracic contusive SCI. **(a)** Open field function of a cohort of mice run in replication of the original study was assessed using the BMS and scored by two blinded observers. There was no difference between *Myrf* ICKO and control on the BMS following thoracic SCI (*F (1, 17)=0.017, P=0.899*). **(b)** A graph of the BMS subscore also shows no differences between *Myrf* ICKO and controls (*F(1, 17)=0.003, P=0.960*). **(c)** Graph demonstrating the percentage of hindlimb errors (error / error + success) on the regular horizontal ladder task. Following injury, there is no statistical difference between groups (*F(1, 15) = 1.772, P=0.203*). **(d-i)** Catwalk analysis was used to determine differences in various parameters of gait following thoracic SCI. No difference between *Myrf* ICKO and controls was detected in **(d)** stride length (*F (1, 17)=0.415*, *P=0.528*), **(e)** hindlimb base of support (*F(1, 17)=2.262, P=0.151*), **(f)** paw position (*F(1, 17)=0.556, P=0.466*) **(g)** hindlimb duty cycle (*F(1, 17)= 0.921, P=0.351*) **(h)** percent of run with 1 or 2 paws on the ground (*F(1, 17)= 0.679, P=0.421*), and **(i)** percent of run with 3 or 4 paws on the ground (*F(1, 17)=0.439*, *P=0.517*). Groups were compared at all time points post injury. All statistical comparisons were made using a two-way repeated measures ANOVA. Individual time points were compared with Šídák *post hoc* test. ns = non-significant. Bars are mean ± SEM.

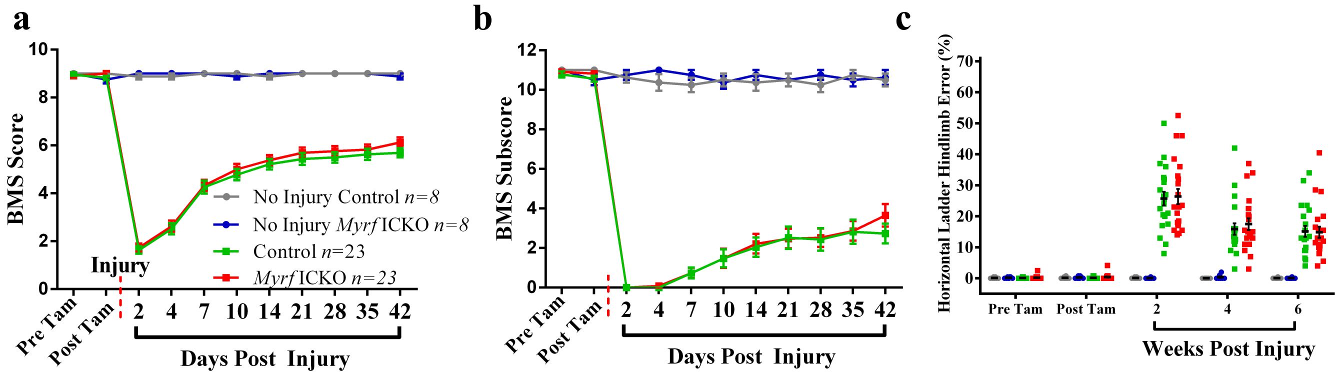


**Supplementary Fig. 2**

Compiled data from both cohorts reveals no difference between control and *Myrf* ICKO mice in locomotor recovery following thoracic SCI. **(a)** Graph of open field BMS score reveals no differences between *Myrf* ICKO and control mice with an injury (*F (8, 464) = 80.47, P<0.001; injured Myrf ICKO vs injured control P=0.783*) or on **(B)** BMS subscore (*F(3, 58)=148.7, P<0.001, injured Myrf ICKO vs injured control P=0.988*). **(C)** Graph of the assessment of horizontal regular ladder errors (% = error / error + success) reveals no differences (*F (3, 55)=24.76, P<0.001; injured Myrf ICKO vs control, P=0.999*). Groups were compared post injury. All statistical comparisons were made using a two-way repeated measures ANOVA, and compared with a Tukey's *post hoc* for individual group differences. Error bars are mean +/- SEM.

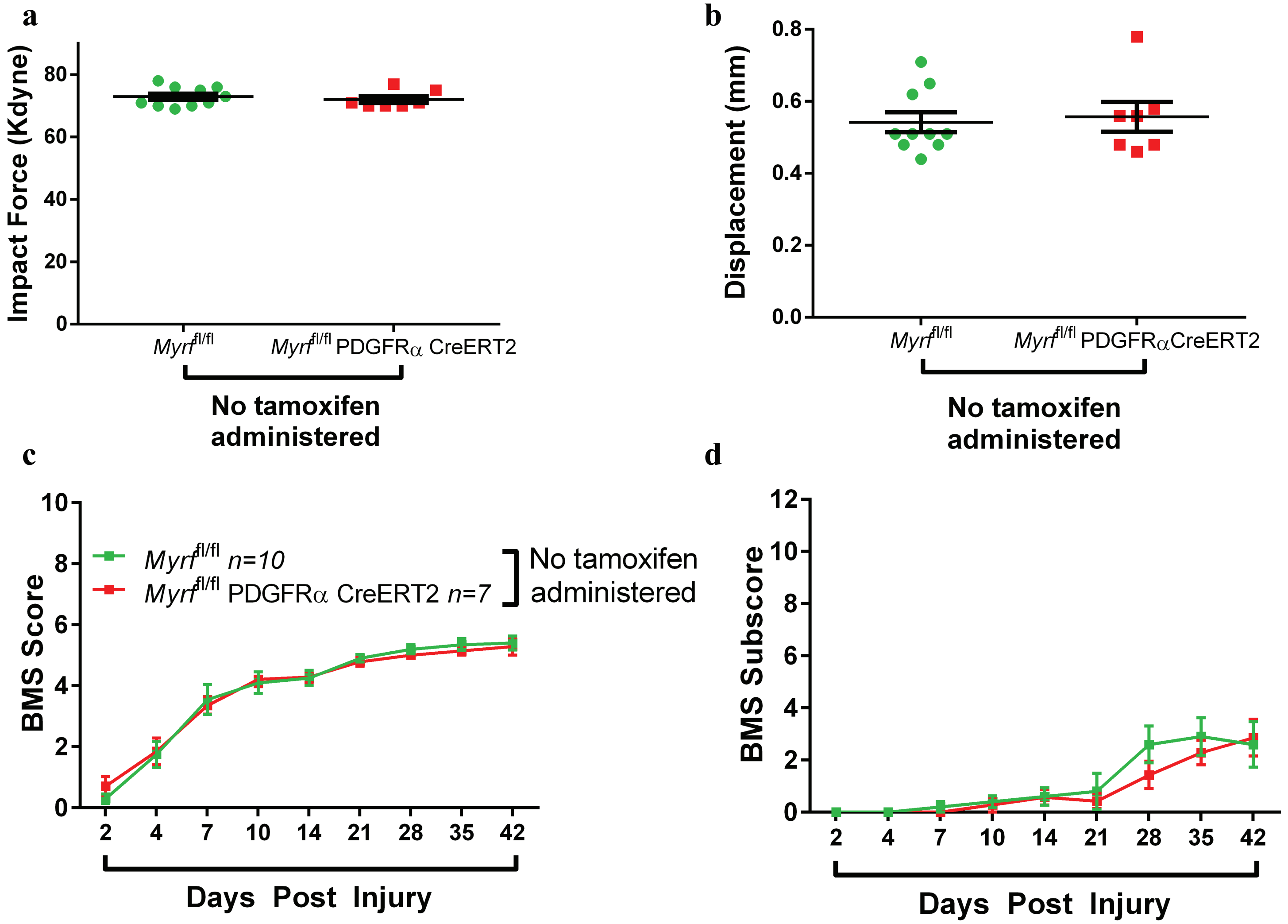


**Supplementary Fig. 3**

*Myrf*$^{fl/fl}$ mice do not inherently differ in locomotor recovery from moderate thoracic SCI relative to *Myrf*$^{fl/fl}$ PDGFRα CreERT2 in the absence of tamoxifen. (**a**) Measurement of impact force imparted on the spinal cord of *Myrf*$^{fl/fl}$ and *Myrf*$^{fl/fl}$ PDGFRα CreERT2 reveals no differences between groups (*df=15, t=0.604, P=0.555, Student's t-test)*. **(b)** A graph of the displacement of the impactor tip into the spinal cord during thoracic contusion indicates no distinction between *Myrf*$^{fl/fl}$ and *Myrf*$^{fl/fl}$ PDGFRα CreERT2 mice (*df=15, t=0.318, P=0.755, Student's t-test)*. Graphs of open field performance as assessed on the **(c)** BMS, and **(d)** BMS subscore indicate no differences in hindlimb recovery between *Myrf*$^{fl/fl}$ and *Myrf*$^{fl/fl}$ PDGFRα CreERT2 in the absence of tamoxifen *(BMS F(1,15)=0.004, P=0.951; BMS subscore F(1,15)=0.371, P=0.552, two-way repeated measures ANOVA)*. Error bars are +/- SEM.
